# Calibration-free speckle matrix imaging

**DOI:** 10.1038/s41377-022-00723-w

**Published:** 2022-02-08

**Authors:** Philipp del Hougne

**Affiliations:** Univ Rennes, CNRS, IETR - UMR 6164, 35000 Rennes, France

**Keywords:** Microscopy, Imaging and sensing, Adaptive optics

## Abstract

Unknown speckle patterns can be used to image targets embedded in complex scattering media 100 times faster than previous techniques based on carefully calibrated illuminations.

## Graphical abstract

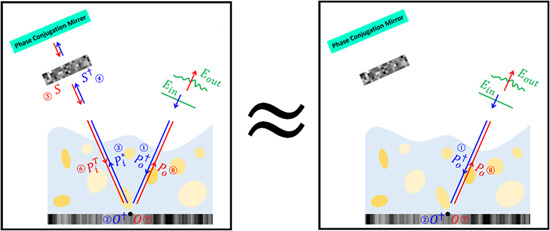

Imaging is the art of retrieving a representation of a scene based on how it scatters waves. When the scene is in free space, this process is conceptually easy; for instance, one can scan a focal spot across the scene. However, in many practical scenarios of interest, the scene is embedded in a heterogeneous environment that distorts wavefronts through local phase retardations (aberration) and/or through multiple scattering. These wavefront distortions are universal challenges faced across all areas of wave imaging, from optical deep-tissue imaging, via ultrasound and seismologic imaging, to microwave-based radar systems.

By sculpting the impinging wavefront, it is possible to focus (and thus image) inside complex scattering media^1^. However, these adaptive-optics approaches usually rely on some kind of guidestar inside the medium to determine the wavefront correction^2^. Noninvasive approaches that do not require sample labeling are highly desired, e.g., for in vivo biological imaging. However, without adapting the input wavefront, the output wavefront is a seemingly arbitrary speckle pattern. Advanced computational approaches do enable noninvasive imaging based on such outputs, but only for isolated objects within the memory effect’s limited range, which decreases with depth^3,4^.

An alternative approach to injecting a plane or optimized wavefront into the system consists of fully characterizing the linear system’s input–output relation and subsequently analyzing it in postprocessing. Indeed, once the matrix containing the Green’s functions between input modes (e.g., pixels of a deformable mirror) and output modes (e.g., pixels of a camera) is known, the linearity of the wave equation allows us to calculate the output wavefront for any desired input wavefront^5^. Such “matrix imaging” goes far beyond a digital implementation of adaptive optics in postprocessing because all ever accessible information about the system given the available input and output channels is now at our disposal. Advanced signal processing techniques can then isolate the effect of “ballistic” waves that were not distorted and directly reveal information about the target’s reflectivity^6–9^. However, the benefits of matrix imaging come at a price: measuring the matrix in the first place is time-consuming, expensive and vulnerable to perturbations.

Now, writing in *Light: Science & Applications*^10^, a team led by Wonshik Choi reports a clever trick for matrix imaging to simultaneously (i) speed up the data acquisition by almost a factor of 100 and (ii) remove the need for carefully characterizing the wavefronts used to measure the matrix. They note that under a few simplifying assumptions, the “time-reversal matrix” (TRM) also enables the identification of ballistic waves with advanced processing tools. While the usual reflection matrix (RM) links input channels (e.g., SLM pixels) to output channels (e.g., camera pixels), the TRM can be understood as linking the output channels to themselves, as illustrated in Fig. 1(b1). Under Choi’s assumptions, the role played by the input channels essentially disappears (Fig. 1(b2)) with two important consequences: first, there is no need to finely sample all input channels and subsequently correct input aberrations; second, the specific choices of input illumination patterns do not matter or need to be known, as long as they are mutually orthogonal.

**Fig. 1:**
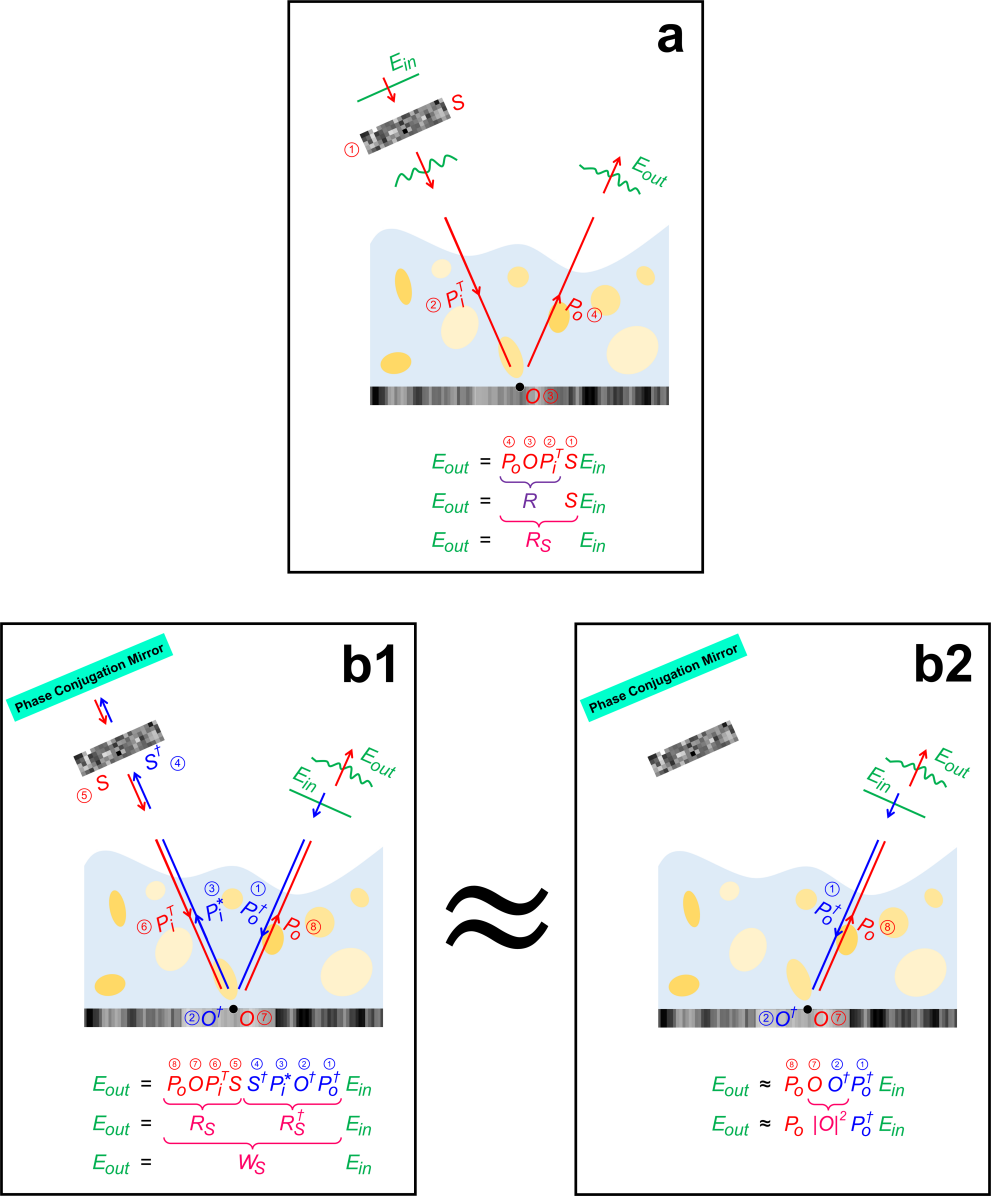
Concept of calibration-free speckle matrix imaging. **a** The conventional approach to matrix imaging is based on the reflection matrix *R* linking the output wavefront *E*_out_ to the input wavefront *E*_in_. The goal is to retrieve the object’s reflectivity *O*. *R* can also be measured in a different basis *S* as *R*_S_ = *RS*. **b1** The time-reversal reflection matrix $W_{\mathrm{S}} = R_{\mathrm{S}}R_{\mathrm{S}}^{\dagger}$ describes the time-reversed process from **a** (blue), followed by the process from **a** (red). **b2** The process from **b1** can be approximated under certain conditions to depend only on the object and propagation between the object and the output detector array. Therefore, unknown speckle illuminations can be used to compressively measure $W_{\mathrm{S}} = R_{\mathrm{S}}R_{\mathrm{S}}^{\dagger}$ with low latency and without explicitly reconstructing *R*.

Choi’s team then leverages the well-known pseudoorthogonality of speckle patterns to generate the necessary illuminations. The team replaces the previously deployed elaborate wavefront-shaping setup on the input side with a simple rotating diffuser that does not require any calibration. Moreover, they notice that because the RM is a very sparse matrix, they need much fewer scene illuminations than there are unknowns in the RM. Thereby, Choi and coworkers combine three distinct ideas in a unique way. On the one hand, *compressed sensing* is well established and routinely used to image a scene from an underdetermined set of measurements. Recently, reconstructing the matrix—as opposed to the scene—from compressive measurements was explored^11^, but Choi’s team does not even need to explicitly reconstruct the compressively sampled RM in their approach. On the other hand, *calibration-free imaging with speckle illumination* is known from blind structured-illumination microscopy^12^ but not in the context of matrix imaging. And, in fact, even the *time-reversal matrix* itself is a well-known operator for blind selective focusing on strong reflectors^13^ and has recently been used in matrix imaging^7^. However, whereas these approaches were based on a singular value decomposition (SVD) of the TRM, Choi’s team expresses the TRM as a matrix product in a way that reveals that calibration-free speckle illumination may work and speed up matrix imaging.

Looking forward, it will be interesting to understand potential conceptual links between Choi’s iterative postprocessing of the TRM and the traditional SVD approach^7^; moreover, it may be possible to refine the cost functional of Choi’s algorithm, for instance, using image quality metrics^14^ and/or techniques that previously enabled “superresolution”^12^. Given that latency improvements are a key motivation for compressive sampling in Choi’s work, it is insightful to note parallels with the role of compressive sensing in microwave computational meta-imaging. While the latter is primarily concerned with scenes in free space, latency is a crucial metric, too, and over the last decade, tremendous progress beyond conventional compressed sensing has been made to unlock remarkable latency improvements through the use of fewer but tailored illumination patterns^15^. Clearly, an orthogonal illumination basis would be an improvement upon the pseudoorthogonality of speckles and reduce reconstruction noise, but—given some a priori knowledge—a series of fewer learned illumination patterns that highlight salient features may yield further remarkable latency improvements^15^. To implement orthogonal or learned illumination patterns, wavefront shaping on the input side with digital micromirror devices could match the camera frame rate of up to 12.5 kHz in Choi’s current work but would add complexity that must be traded off against further latency gains.

On a yet more fundamental level, the treatment of multiple scattering in matrix imaging may need to be revisited in the future. The current approach of discarding it as random noise not only neglects the fact that “multiple-scattering noise”—despite its random appearance upon visual inspection—does contain profound mesoscopic correlations. More intriguing is the fact that multiply scattered waves may actually carry more information about the scene than “ballistic” waves: the sensitivity of waves to subwavelength scene details, and hence the waves’ ability to extract finely resolved scene information, increases with their dwell time^16^. Reverberation and multiple scattering can be thought of as generalizing the deeply subwavelength interferometric sensitivity from a conventional 1D interferometer to arbitrary geometries. However, this precious information is encoded in multiplexed measurements and challenging to decode.

Choi’s team has brought fresh ideas into matrix imaging, making it much simpler and faster, and their work^10^ will undoubtedly trigger the exploration of new avenues in matrix imaging across all wave engineering disciplines.

## References

[1] Vellekoop IM, Mosk AP (2007). Focusing coherent light through opaque strongly scattering media. Opt. Lett..

[2] Horstmeyer R, Ruan HW, Yang C (2015). Guidestar-assisted wavefront-shaping methods for focusing light into biological tissue. Nat. Photonics.

[3] Bertolotti J (2012). Non-invasive imaging through opaque scattering layers. Nature.

[4] Katz O (2014). Non-invasive single-shot imaging through scattering layers and around corners via speckle correlations. Nat. Photonics.

[5] Popoff SM (2010). Measuring the transmission matrix in optics: an approach to the study and control of light propagation in disordered media. Phys. Rev. Lett..

[6] Kang S (2015). Imaging deep within a scattering medium using collective accumulation of single-scattered waves. Nat. Photonics.

[7] Badon A (2016). Smart optical coherence tomography for ultra-deep imaging through highly scattering media. Sci. Adv..

[8] Kang S (2017). High-resolution adaptive optical imaging within thick scattering media using closed-loop accumulation of single scattering. Nat. Commun..

[9] Badon A (2020). Distortion matrix concept for deep optical imaging in scattering media. Sci. Adv..

[10] Lee H (2022). High-throughput volumetric adaptive optical imaging using compressed time-reversal matrix. Light Sci. Appl..

[11] Li SH (2021). Compressively sampling the optical transmission matrix of a multimode fibre. Light Sci. Appl..

[12] Mudry E (2012). Structured illumination microscopy using unknown speckle patterns. Nat. Photonics.

[13] Prada C, Fink M (1994). Eigenmodes of the time reversal operator: a solution to selective focusing in multiple-target media. Wave Motion.

[14] Yeminy T, Katz O (2021). Guidestar-free image-guided wavefront shaping. Sci. Adv..

[15] Saigre-Tardif, C. et al. Intelligent meta-imagers: from compressed to learned sensing. *Appl. Phys. Rev.* in press (2022), preprint at https://arxiv.org/abs/2110.14022v3.

[16] del Hougne M, Gigan S, del Hougne P (2021). Deeply subwavelength localization with reverberation-coded aperture. Phys. Rev. Lett..

