# Publisher Correction: Calibration-free speckle matrix imaging

**DOI:** 10.1038/s41377-022-00735-6

**Published:** 2022-03-03

**Authors:** Philipp del Hougne

**Affiliations:** Univ Rennes, CNRS, IETR - UMR 6164, 35000 Rennes, France

**Keywords:** Microscopy, Imaging and sensing, Adaptive optics

Correction to: *Light: Science & Applications*

10.1038/s41377-022-00723-w published online 8 February 2022

After publication of this article^1^, it is reported that the Abstract is missing. The Abstract is provided below:

Unknown speckle patterns can be used to image targets embedded in complex scattering media 100 times faster than previous techniques based on carefully calibrated illuminations.

The original article has been updated.
